# Biofilm formation by staphylococci and streptococci: structural, functional, and regulatory aspects and implications for pathogenesis

**DOI:** 10.3389/fcimb.2015.00031

**Published:** 2015-04-08

**Authors:** Pietro Speziale, Joan A. Geoghegan

**Affiliations:** ^1^Unit of Biochemistry, Department of Molecular Medicine, Università Degli Studi di PaviaPavia, Italy; ^2^Department of Microbiology, School of Genetics and Microbiology, Moyne Institute of Preventive Medicine, Trinity College DublinDublin, Ireland

**Keywords:** Staphylococcus, Streptococcus, biofilm, structure, regulation, pathogenesis

Members of the genus *Staphylococcus* and *Streptococcus* are commensals and opportunistic pathogens that cause a variety of infections in human and animals. Biofilm formation is an important survival strategy adopted by these Gram-positive cocci. Biofilms are surface-associated, specialized multicellular bacterial communities embedded in a self-produced matrix of polysaccharides, proteins, DNA or combinations thereof. Biofilm growth allows bacteria to persist *in vivo* by resisting host immune defenses and antibiotics meaning that these infections are difficult to eradicate and treat clinically. Biofilms form in many natural niches occupied by staphylococci and streptococci (such as the nasopharynx, heart valves, lungs, and oral cavity) and are important during the infection of implanted medical devices.

Biofilms develop in multiple stages to form highly ordered multicellular communities (O'Toole et al., 2000; Otto, 2013). Initially the bacteria must adhere to a surface or host tissue (primary attachment phase) before they proliferate to form multicellular aggregates (accumulation phase). During the maturation stage, channels and mushroom-shaped structures are created to allow nutrients to penetrate the deeper layers of the biofilm (O'Toole et al., 2000). Finally, during the dispersal stage, bacteria detach from the biofilm and disseminate to new sites (Otto, 2013).

In this series of articles, the authors provide an overview of the molecular components involved in biofilm formation by staphylococci and streptococci. Particular emphasis is placed on the mechanisms of development and dispersal of biofilm and the regulation of biofilm formation. Novel therapeutic strategies for the prevention and eradication of medical biofilms are also discussed.

Arciola et al. (2015) describe our evolving understanding of the enzymes involved in the biosynthesis of polysaccharide intracellular adhesion (PIA), a polymer that mediates staphylococcal biofilm accumulation. They discuss the genetic control of factors involved in biofilm formation and disruption. McCarthy et al. (2015) explore the different mechanisms used by methicillin-sensitive *Staphylococcus aureus* (MSSA) and methicillin-resistant *S. aureus* (MRSA) to form biofilm and the impact of methicillin resistance on PIA production and virulence *in vivo*. Speziale et al. (2014) describe proteins of staphylococci that contribute to primary attachment and biofilm accumulation. The current understanding of the mechanistic basis of cell wall anchored surface protein-dependent biofilm accumulation is described. Büttner et al. (2015) capture recent advances in our understanding of the contribution of *Staphylococcus epidermidis* multifunctional surface proteins to the primary attachment and accumulation phases of biofilm formation. They also illustrate the integration of biofilm-promoting factors into regulatory networks and emphasize how these factors contribute to the adaptation of *S. epidermidis* to changing environmental conditions. The review by Le et al. (2014) presents evidence of the critical activity of phenol soluble modulins (PSMs) as mediators of biofilm structuring and dispersal, stressing their role in the formation of biofilm channels and dissemination of clusters of biofilm to distal organs *in vivo*. In keeping with the theme of dispersal, Lister and Horswill (2014) report the current understanding of enzymatic dispersal mechanisms (proteases, nucleases, and dispersin B) and the newly described broad-spectrum effects of D-amino acids and synthetic cationic peptides.

Gilley and Orihuela (2014) describe the reasons why biofilm-forming *Streptococcus pneumoniae* colonizing the nasopharynx of humans are not invasive. The bacteria in the biofilm have a decreased rate of metabolism, produce less capsular polysaccharide and less pneumolysin than their planktonic counterparts. The authors suggest that this may explain long-term, asymptomatic nasopharyngeal colonization by *S. pneumoniae* in humans. Chao et al. (2015) describe the increased propensity of *S. pneumoniae* in a biofilm to exchange genetic material. Mechanisms involved in dispersal during colonization and disease are also considered.

Fiedler et al. (2015) review the clinical relevance of biofilm formation by the human pathogen *Streptococcus pyogenes*, in particular during oto-nasopharyngeal and skin infection. Moreover, they report the involvement of capsule carbohydrates, pili, surface proteins, and secreted enzymes and the regulatory aspects of the biofilm lifestyle of *S. pyogenes*. Rosini and Margarit (2015) discuss the role of pili and other surface components and the influence of environmental conditions on biofilm production by *Streptococcus agalactiae*. Klein et al. (2015) present evidence of how *Streptococcus mutans* assembles a cariogenic biofilm in the mouth and how extracellular polysaccharide provides mechanical stability and facilitates the creation of highly acidic microenvironments inside the matrix. Extracellular DNA enhances the local synthesis of extracellular polysaccharide, increases the adherence of *S. mutans* to saliva-coated apatitic surfaces and increases the cohesive properties of biofilm.

In conclusion, these reviews summarize one of the most vital areas of research in the pathogenesis of staphylococcal and streptococcal infections. The limitations of *in vitro* techniques, and the urgent need to advance our understanding of biofilm formation using *in vivo* models, are considered. The knowledge generated from this research has the potential to lead to novel approaches to diagnosing and treating biofilm-related infections, and it lays the groundwork for deeper investigation into the basic biology of these diseases.

## Conflict of interest statement

The authors declare that the research was conducted in the absence of any commercial or financial relationships that could be construed as a potential conflict of interest.
